# (*E*)-2-{[(2-(Trifluoro­meth­yl)phen­yl]imino­meth­yl}phenol

**DOI:** 10.1107/S1600536812003212

**Published:** 2012-02-04

**Authors:** Hakkı Yasin Odabaşoğlu, Orhan Büyükgüngör, Osman Ozan Avinç, Mustafa Odabaşoğlu

**Affiliations:** a Department of Textile Engineering, Faculty of Engineering, Pamukkale University, TR-20070 Kınıklı Denizli, Turkey; b Department of Physics, Faculty of Arts and Science, Ondokuz Mayıs University, TR-55139 Kurupelit Samsun, Turkey; c Department of Chemical Technonolgy, Pamukkale University, TR-20070 Kınıklı Denizli, Turkey

## Abstract

In the crystal of the title compound, C_14_H_10_F_3_NO, intra­molecular O—H⋯N and O—H⋯F hydrogen bonds generate *S*(6) and *S*(10) intramolecular hydrogen-bonded rings. The dihedral angle between the planes of the aromatic rings is 13.00 (14)°.

## Related literature

For related structures, see: Odabaşoǧlu *et al.* (2003 ▶, 2005 ▶); Albayrak *et al.* (2012 ▶); Temel *et al.* (2006 ▶). For ring motifs, see: Bernstein *et al.* (1995 ▶). For azomethine dye applications, see: Williams (1972 ▶); Elizbarashvili *et al.* (2007 ▶); Taggi *et al.* (2002 ▶); Ichijima & Kobayashi (2005 ▶); Calligaris *et al.* (1972 ▶); Hadjoudis *et al.* (1987 ▶). For the synthesis of the title mol­ecule, see: Odabaşoǧlu *et al.* (2003 ▶).
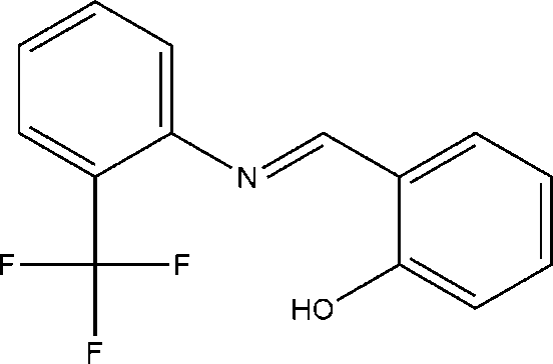


## Experimental

### 

#### Crystal data

C_14_H_10_F_3_NO
*M* *_r_* = 265.23
Orthorhombic, 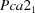

*a* = 17.9907 (18) Å
*b* = 5.0898 (4) Å
*c* = 13.2564 (10) Å
*V* = 1213.88 (18) Å^3^
*Z* = 4
Mo *K*α radiation
μ = 0.12 mm^−1^
*T* = 296 K
0.73 × 0.48 × 0.27 mm

#### Data collection

Stoe IPDS II diffractometer
Absorption correction: integration (*X-RED32*; Stoe & Cie, 2002 ▶) *T*_min_ = 0.932, *T*_max_ = 0.966
9185 measured reflections
2519 independent reflections
1827 reflections with *I* > 2σ(*I*)
*R*_int_ = 0.038

#### Refinement

*R*[*F* ^2^ > 2σ(*F* ^2^)] = 0.037
*wR*(*F* ^2^) = 0.084
*S* = 1.07
1318 reflections
176 parameters
1 restraint
H atoms treated by a mixture of independent and constrained refinement
Δρ_max_ = 0.11 e Å^−3^
Δρ_min_ = −0.15 e Å^−3^
Absolute structure: 1201 measured Friedel pairs were merged, because the compound is a weak anomalous scatterer

### 

Data collection: *X-AREA* (Stoe & Cie, 2002 ▶); cell refinement: *X-AREA*; data reduction: *X-RED32* (Stoe & Cie, 2002 ▶); program(s) used to solve structure: *SHELXS97* (Sheldrick, 2008 ▶); program(s) used to refine structure: *SHELXL97* (Sheldrick, 2008 ▶); molecular graphics: *ORTEP-3* (Farrugia, 1997 ▶); software used to prepare material for publication: *WinGX* (Farrugia, 1999 ▶).

## Supplementary Material

Crystal structure: contains datablock(s) I, global. DOI: 10.1107/S1600536812003212/bh2411sup1.cif

Structure factors: contains datablock(s) I. DOI: 10.1107/S1600536812003212/bh2411Isup2.hkl

Additional supplementary materials:  crystallographic information; 3D view; checkCIF report

## Figures and Tables

**Table 1:** Hydrogen-bond geometry (Å, °)

*D*—H⋯*A*	*D*—H	H⋯*A*	*D*⋯*A*	*D*—H⋯*A*
O1—H13⋯N1	0.82 (5)	1.88 (5)	2.622 (3)	149 (4)
O1—H13⋯F3	0.82 (5)	2.58 (4)	3.179 (3)	131 (4)

**Fig. 1.**
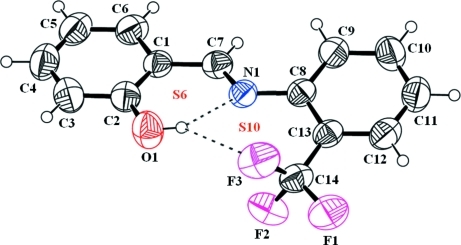
A view of the title molecule with displacement ellipsoids at the 40% probability level.

**Fig. 2.**
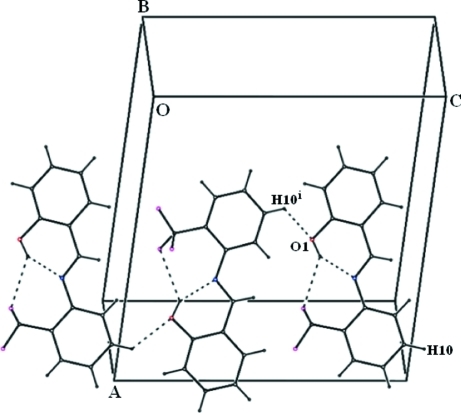
Part of the crystal structure, showing the formation of S(6), S(10) rings and C(9) chain motifs. H atoms not involved in hydrogen bonds have been omitted for clarity [Symmetry code: (i) 1 - x, y - 1, z - 1/2].

**Fig. 3.**
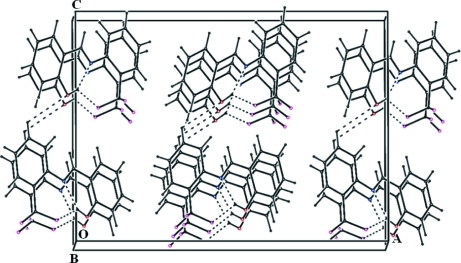
A packing diagram with hydrogen bonds drawn as dashed lines. H atoms not involved in hydrogen bonds have been omitted for clarity.

## supplementary crystallographic information

### Comment

Most azmethine dyes are known to have biological activities and have been used as antimicrobial, antifungal, antitumor, antitoxic, anti-inflammatory and as herbicides (Williams, 1972; Elizbarashvili *et al.*, 2007). In industry, they have a wide range of applications such as dyes and pigments with luminescent properties (Taggi *et al.*, 2002). Azmethine dyes are known to be among the most important dyes because of their wide applications, including color photographic systems, dye diffusion thermal transfer print systems and others (Ichijima & Kobayashi, 2005). In addition, azmethine dyes have been used widely as ligands in the field of coordination chemistry (Calligaris *et al.*, 1972). *o*-Hydroxy azmethine dyes are characterized by a strong intramolecular hydrogen bond. These compounds are of interest because of their thermochromism and photochromism in the solid state, which can involve reversible intramolecular proton transfer from an O atom to the neighboring N atom. It was proposed on the basis of thermochromic and photochromic azmethine dyes that the molecules exhibiting thermochromism are planar while the molecules exhibiting photochromism are non-planar (Hadjoudis *et al.*, 1987). Taking in account these important features of the *o*-hydroxy azomethine dyes, we aimed to investigate the intra- and/or intermolecular interactions and the conformation of the title compound, (*E*)-2-[(2-(trifluoromethyl)phenylimino)methyl]phenol, by X-ray crystallography.

*o*-Hydroxy azomethine dyes can exist in three tautomeric structures, as enol (Odabaşoǧlu *et al.*, 2005), keto (Albayrak *et al.*, 2012) and zwitterionic (Temel *et al.*, 2006) forms in the solid state. In the title compound, the enol tautomer is favoured over the keto and zwitterionic forms (Fig. 1 and Table 1), and there is an intramolecular O1—H13···N1 hydrogen bond (Table 2). The molecule is almost planar, with a dihedral angle of 13.00 (14)° between C1···C6 and C8···C13 rings. The O—H···N hydrogen-bonded ring is planar and is coupled with the phenylene ring [dihedral angle is 0.8 (6)°]. The crystal packing is stabilized by O—H···N, O—H···F and C—H···O hydrogen bond interactions. O1—H13···N1 and O1—H13···F3 hydrogen bonds generate *S*(10) ring motif which includes the *S*(6) ring (Bernstein *et al.*, 1995). In addition, C10—H10···O1 hydrogen bond generate *C*(9) chain (Fig. 2) and a three-dimensional network (Fig. 3).

### Experimental

The title molecule was prepared as described by Odabaşoǧlu *et al.* (2003), using 2-trifluoromethylaniline and salicylaldehyde as starting materials. Crystals were obtained from an ethyl alcohol solution by slow evaporation (yield 92%, m.p. 338 K).

### Refinement

H atom bonded to O1 was located in a difference map and refined isotropically. Constrained bond lengths and isotropic *U* parameters for aromatic C—H: 0.93 Å and *U*_iso_(H) = 1.2*U*_eq_(C).

### Crystal data

**Table:**

C_14_H_10_F_3_NO	*D*_x_ = 1.451 Mg m^−^^3^
*M**_r_* = 265.23	Melting point: 338 K
Orthorhombic, *P**c**a*2_1_	Mo *K*α radiation, λ = 0.71073 Å
Hall symbol: P 2c -2ac	Cell parameters from 15262 reflections
*a* = 17.9907 (18) Å	θ = 1.5–28.0°
*b* = 5.0898 (4) Å	µ = 0.12 mm^−^^1^
*c* = 13.2564 (10) Å	*T* = 296 K
*V* = 1213.88 (18) Å^3^	Prism, yellow
*Z* = 4	0.73 × 0.48 × 0.27 mm
*F*(000) = 544	

### Data collection

**Table:**

Stoe IPDS II diffractometer	2519 independent reflections
Radiation source: fine-focus sealed tube	1827 reflections with *I* > 2σ(*I*)
Graphite monochromator	*R*_int_ = 0.038
Detector resolution: 0 pixels mm^-1^	θ_max_ = 26.5°, θ_min_ = 2.3°
rotation method scans	*h* = −22→22
Absorption correction: integration (*X-RED32*; Stoe & Cie, 2002)	*k* = −6→6
*T*_min_ = 0.932, *T*_max_ = 0.966	*l* = −16→16
9185 measured reflections	

### Refinement

**Table:**

Refinement on *F*^2^	Primary atom site location: structure-invariant direct methods
Least-squares matrix: full	Secondary atom site location: difference Fourier map
*R*[*F*^2^ > 2σ(*F*^2^)] = 0.037	Hydrogen site location: inferred from neighbouring sites
*wR*(*F*^2^) = 0.084	H atoms treated by a mixture of independent and constrained refinement
*S* = 1.07	*w* = 1/[σ^2^(*F*_o_^2^) + (0.0476*P*)^2^] where *P* = (*F*_o_^2^ + 2*F*_c_^2^)/3
1318 reflections	(Δ/σ)_max_ < 0.001
176 parameters	Δρ_max_ = 0.11 e Å^−^^3^
1 restraint	Δρ_min_ = −0.15 e Å^−^^3^
0 constraints	Absolute structure: 1201 measured Friedel pairs were merged, because the compound is a weak anomalous scatterer

### Fractional atomic coordinates and isotropic or equivalent isotropic displacement parameters (Å^2^)

**Table:**

	*x*	*y*	*z*	*U*_iso_*/*U*_eq_	
C1	0.45358 (12)	1.0002 (5)	0.7729 (2)	0.0617 (6)	
C2	0.43472 (14)	1.0230 (5)	0.6716 (2)	0.0698 (7)	
C3	0.38195 (19)	1.2075 (6)	0.6431 (3)	0.0864 (9)	
H3	0.3689	1.2237	0.5755	0.104*	
C4	0.34888 (18)	1.3658 (6)	0.7134 (4)	0.0915 (11)	
H4	0.3140	1.4894	0.6928	0.110*	
C5	0.36636 (17)	1.3453 (6)	0.8136 (3)	0.0868 (10)	
H5	0.3433	1.4530	0.8608	0.104*	
C6	0.41827 (16)	1.1637 (6)	0.8435 (3)	0.0793 (8)	
H6	0.4302	1.1486	0.9115	0.095*	
C7	0.50948 (15)	0.8157 (5)	0.8071 (2)	0.0648 (6)	
H7	0.5213	0.8108	0.8753	0.078*	
C8	0.59564 (12)	0.4792 (5)	0.7836 (2)	0.0612 (6)	
C9	0.60401 (16)	0.4129 (6)	0.8843 (3)	0.0780 (8)	
H9	0.5728	0.4895	0.9319	0.094*	
C10	0.65715 (18)	0.2370 (7)	0.9161 (3)	0.0885 (9)	
H10	0.6627	0.2004	0.9844	0.106*	
C11	0.70208 (16)	0.1155 (6)	0.8462 (3)	0.0891 (10)	
H11	0.7380	−0.0038	0.8672	0.107*	
C12	0.69381 (17)	0.1702 (5)	0.7458 (3)	0.0811 (9)	
H12	0.7235	0.0845	0.6987	0.097*	
C13	0.64133 (14)	0.3532 (5)	0.7135 (2)	0.0644 (7)	
C14	0.63575 (17)	0.4148 (6)	0.6040 (2)	0.0769 (8)	
N1	0.54272 (12)	0.6611 (4)	0.74774 (15)	0.0622 (5)	
O1	0.46592 (15)	0.8714 (5)	0.60028 (16)	0.0918 (7)	
F1	0.68225 (14)	0.2715 (5)	0.54915 (16)	0.1234 (8)	
F2	0.65111 (13)	0.6642 (4)	0.58376 (16)	0.1042 (7)	
F3	0.56880 (12)	0.3721 (4)	0.56487 (15)	0.1069 (7)	
H13	0.496 (3)	0.776 (8)	0.628 (4)	0.107 (14)*	

### Atomic displacement parameters (Å^2^)

**Table:**

	*U*^11^	*U*^22^	*U*^33^	*U*^12^	*U*^13^	*U*^23^
C1	0.0553 (12)	0.0619 (14)	0.0680 (16)	−0.0060 (11)	0.0034 (13)	0.0022 (13)
C2	0.0679 (15)	0.0666 (15)	0.0750 (18)	−0.0014 (13)	−0.0018 (15)	0.0004 (15)
C3	0.087 (2)	0.0808 (19)	0.091 (2)	0.0044 (16)	−0.0121 (18)	0.0104 (18)
C4	0.0713 (18)	0.0710 (16)	0.132 (4)	0.0094 (15)	−0.001 (2)	0.005 (2)
C5	0.0714 (18)	0.0773 (19)	0.112 (3)	0.0054 (15)	0.0153 (18)	−0.0065 (18)
C6	0.0755 (17)	0.0786 (17)	0.084 (2)	0.0003 (15)	0.0129 (16)	−0.0033 (17)
C7	0.0632 (14)	0.0720 (15)	0.0593 (14)	−0.0052 (13)	0.0005 (12)	−0.0027 (13)
C8	0.0525 (12)	0.0627 (14)	0.0683 (16)	−0.0069 (11)	−0.0001 (13)	−0.0031 (13)
C9	0.0711 (16)	0.090 (2)	0.0725 (18)	0.0070 (15)	0.0017 (14)	0.0019 (17)
C10	0.085 (2)	0.092 (2)	0.088 (2)	0.0053 (18)	−0.0119 (19)	0.0170 (18)
C11	0.0669 (17)	0.0803 (19)	0.120 (3)	0.0050 (14)	−0.008 (2)	0.016 (2)
C12	0.0658 (16)	0.0713 (16)	0.106 (3)	−0.0003 (15)	0.0126 (16)	−0.0036 (18)
C13	0.0545 (13)	0.0611 (13)	0.0774 (17)	−0.0084 (12)	0.0061 (12)	−0.0056 (14)
C14	0.0745 (17)	0.0743 (18)	0.082 (2)	−0.0008 (14)	0.0190 (16)	−0.0080 (16)
N1	0.0569 (11)	0.0677 (11)	0.0618 (13)	0.0011 (10)	0.0009 (9)	−0.0008 (11)
O1	0.1086 (16)	0.0980 (16)	0.0688 (14)	0.0250 (14)	−0.0129 (12)	−0.0076 (12)
F1	0.1361 (18)	0.1374 (17)	0.0967 (15)	0.0357 (14)	0.0433 (14)	−0.0107 (13)
F2	0.1301 (16)	0.0950 (13)	0.0874 (13)	−0.0185 (11)	0.0113 (11)	0.0147 (10)
F3	0.0988 (13)	0.1415 (18)	0.0803 (12)	−0.0191 (11)	−0.0043 (10)	−0.0233 (14)

### Geometric parameters (Å, º)

**Table:**

C1—C2	1.390 (4)	C8—C13	1.397 (4)
C1—C6	1.404 (4)	C8—N1	1.411 (3)
C1—C7	1.449 (4)	C9—C10	1.376 (4)
C2—O1	1.343 (4)	C9—H9	0.9300
C2—C3	1.388 (4)	C10—C11	1.376 (5)
C3—C4	1.369 (5)	C10—H10	0.9300
C3—H3	0.9300	C11—C12	1.368 (5)
C4—C5	1.368 (6)	C11—H11	0.9300
C4—H4	0.9300	C12—C13	1.394 (4)
C5—C6	1.372 (5)	C12—H12	0.9300
C5—H5	0.9300	C13—C14	1.488 (5)
C6—H6	0.9300	C14—F2	1.326 (3)
C7—N1	1.263 (3)	C14—F1	1.327 (4)
C7—H7	0.9300	C14—F3	1.329 (4)
C8—C9	1.385 (4)	O1—H13	0.82 (5)
			
C2—C1—C6	119.0 (2)	C10—C9—C8	121.9 (3)
C2—C1—C7	121.7 (2)	C10—C9—H9	119.0
C6—C1—C7	119.3 (3)	C8—C9—H9	119.0
O1—C2—C3	118.9 (3)	C9—C10—C11	119.7 (3)
O1—C2—C1	122.0 (2)	C9—C10—H10	120.2
C3—C2—C1	119.1 (3)	C11—C10—H10	120.2
C4—C3—C2	120.7 (4)	C12—C11—C10	120.0 (3)
C4—C3—H3	119.7	C12—C11—H11	120.0
C2—C3—H3	119.7	C10—C11—H11	120.0
C5—C4—C3	121.1 (3)	C11—C12—C13	120.6 (3)
C5—C4—H4	119.5	C11—C12—H12	119.7
C3—C4—H4	119.5	C13—C12—H12	119.7
C4—C5—C6	119.2 (3)	C12—C13—C8	120.1 (3)
C4—C5—H5	120.4	C12—C13—C14	119.1 (3)
C6—C5—H5	120.4	C8—C13—C14	120.8 (2)
C5—C6—C1	121.0 (3)	F2—C14—F1	106.5 (2)
C5—C6—H6	119.5	F2—C14—F3	105.4 (3)
C1—C6—H6	119.5	F1—C14—F3	105.5 (3)
N1—C7—C1	122.5 (2)	F2—C14—C13	112.6 (3)
N1—C7—H7	118.7	F1—C14—C13	112.1 (3)
C1—C7—H7	118.7	F3—C14—C13	114.0 (2)
C9—C8—C13	117.7 (2)	C7—N1—C8	121.2 (2)
C9—C8—N1	123.9 (2)	C2—O1—H13	107 (3)
C13—C8—N1	118.3 (2)		
			
C6—C1—C2—O1	−179.5 (2)	C10—C11—C12—C13	1.5 (5)
C7—C1—C2—O1	1.6 (4)	C11—C12—C13—C8	−1.2 (4)
C6—C1—C2—C3	0.4 (4)	C11—C12—C13—C14	177.8 (3)
C7—C1—C2—C3	−178.6 (3)	C9—C8—C13—C12	−0.8 (3)
O1—C2—C3—C4	−180.0 (3)	N1—C8—C13—C12	−179.4 (2)
C1—C2—C3—C4	0.2 (5)	C9—C8—C13—C14	−179.8 (3)
C2—C3—C4—C5	−0.6 (5)	N1—C8—C13—C14	1.7 (3)
C3—C4—C5—C6	0.4 (5)	C12—C13—C14—F2	−117.6 (3)
C4—C5—C6—C1	0.2 (4)	C8—C13—C14—F2	61.4 (3)
C2—C1—C6—C5	−0.6 (4)	C12—C13—C14—F1	2.5 (4)
C7—C1—C6—C5	178.4 (2)	C8—C13—C14—F1	−178.5 (2)
C2—C1—C7—N1	−1.9 (4)	C12—C13—C14—F3	122.3 (3)
C6—C1—C7—N1	179.2 (2)	C8—C13—C14—F3	−58.7 (3)
C13—C8—C9—C10	2.5 (4)	C1—C7—N1—C8	−178.5 (2)
N1—C8—C9—C10	−179.0 (3)	C9—C8—N1—C7	14.7 (4)
C8—C9—C10—C11	−2.2 (5)	C13—C8—N1—C7	−166.9 (2)
C9—C10—C11—C12	0.1 (5)		

### Hydrogen-bond geometry (Å, º)

**Table:**

*D*—H···*A*	*D*—H	H···*A*	*D*···*A*	*D*—H···*A*
O1—H13···N1	0.82 (5)	1.88 (5)	2.622 (3)	149 (4)
O1—H13···F3	0.82 (5)	2.58 (4)	3.179 (3)	131 (4)
C10—H10···O1^i^	0.93	2.80	3.342 (5)	118

Symmetry code: (i) −*x*+1, −*y*+1, *z*+1/2.
